# Supplementary material for: The mouse neonatal small intestine is regionally specialized for protein absorption and transepithelial transport
Source: Development. 2025 Dec 10;152(23):dev205127. doi: 10.1242/dev.205127 (PMC12746077; doi:10.1242/dev.205127)
Supplement: Supplementary information [file develop-152-205127-s1.pdf]

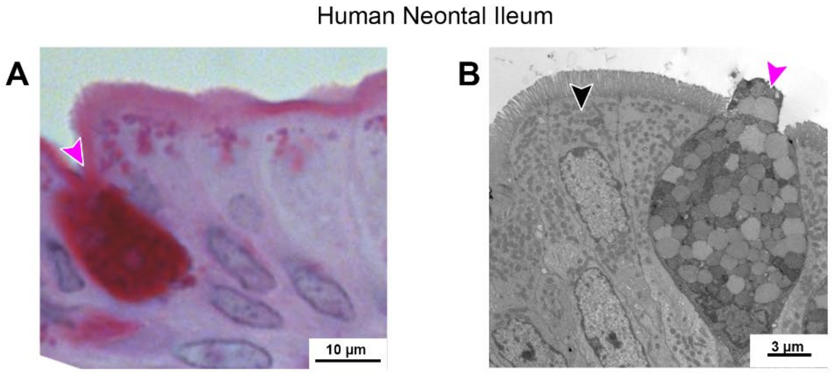

Fig. S1. Goblet cell identification in the human intestine

(A) PAS staining in the ileum of a human patient sample (~58 weeks postmenstrual age), goblet cells can be identified by their deep magenta staining (magenta arrowhead).

(B) Electron micrograph from the ileum of a human patient sample (~58 weeks postmenstrual age). Goblet cells can be distinguished from enterocytes (black arrowhead) by their rich mucin content (magenta arrowhead).

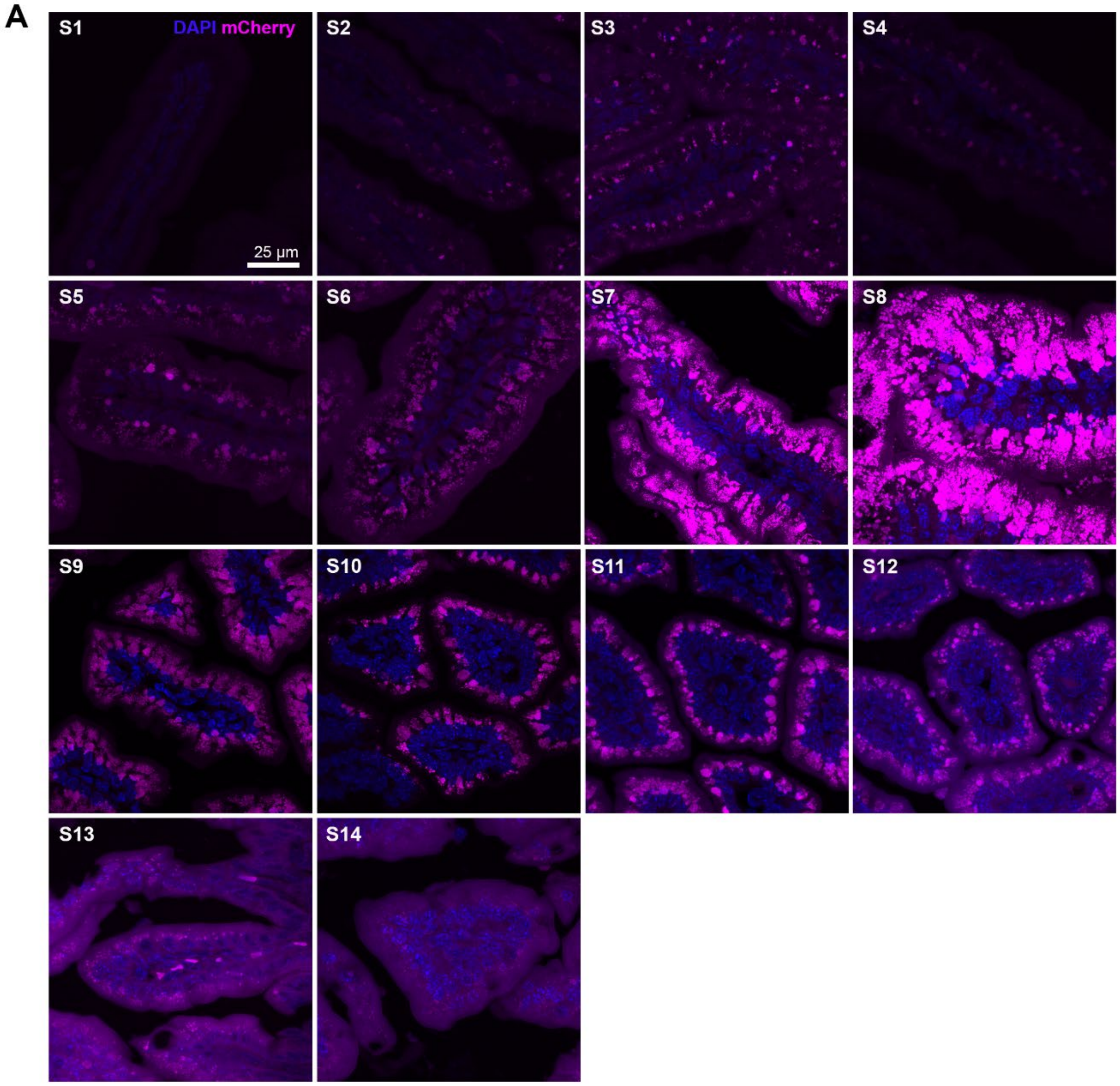

Fig. S2. Regional mCherry uptake

(A) Confocal images from the small intestine of a P6 mouse 3 hours post gavage of mCherry. The mCherry signal is dim through the first four segments and begins to get increasingly brighter through s8 and maintains a bright signal that begins to diminish at S13. Images S1, S7 and S10 are also displayed in Fig. 1F as part of a representation of broader regional differences.

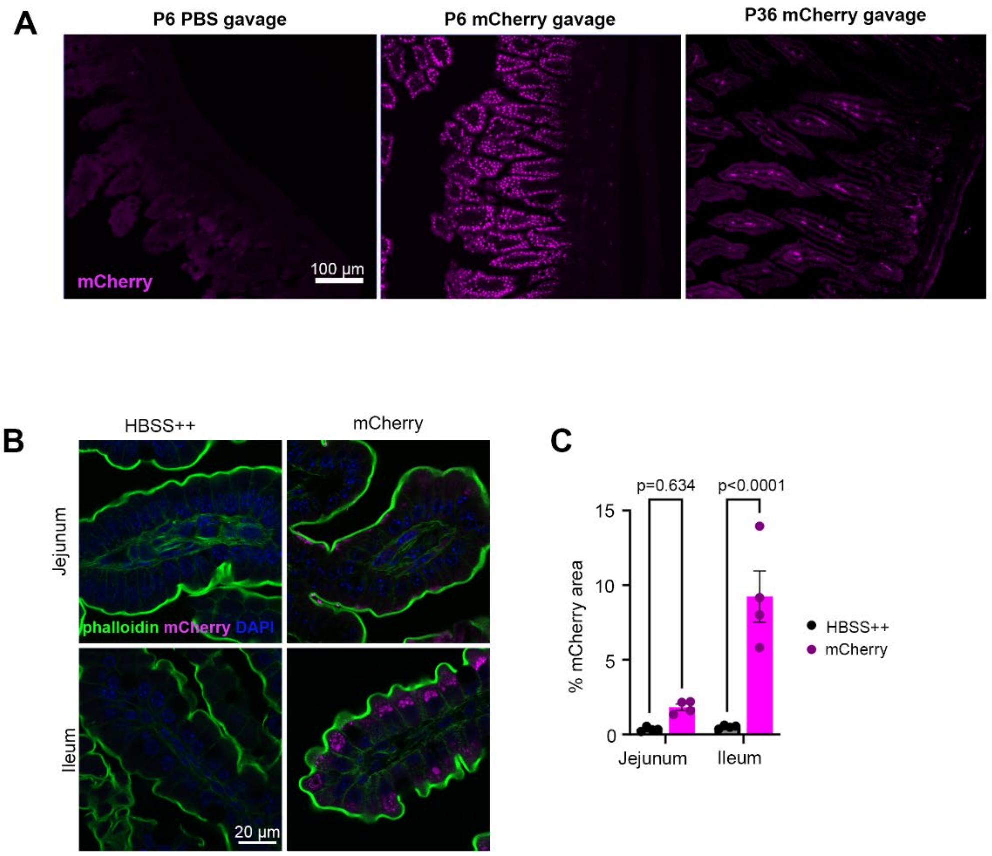

Fig. S3. Minimal autofluorescence in control gavage or ex vivo assays

(A) Confocal images from the ileum of a P6 mouse gavaged with mCherry (left), PBS (middle) or a P36 mouse gavaged with mCherry (right). There is minimal autofluorescence detected in P6 mice gavaged with PBS. Although there is autofluorescence in P36 mice gavaged with mCherry, this signal is largely in the interstitium, which does not have enterocytes.

(B) Representative confocal images of the ex vivo uptake assay. In control samples the lumen was filled with HBSS++, no autofluorescence was detected in these samples.

(C) Quantification of mCherry signal in the ex vivo uptake assay in control (HBSS++) and mCherry conditions. n=4 animals/condition. Two-way ANOVA, post hoc Tukey's multiple comparisons tests.

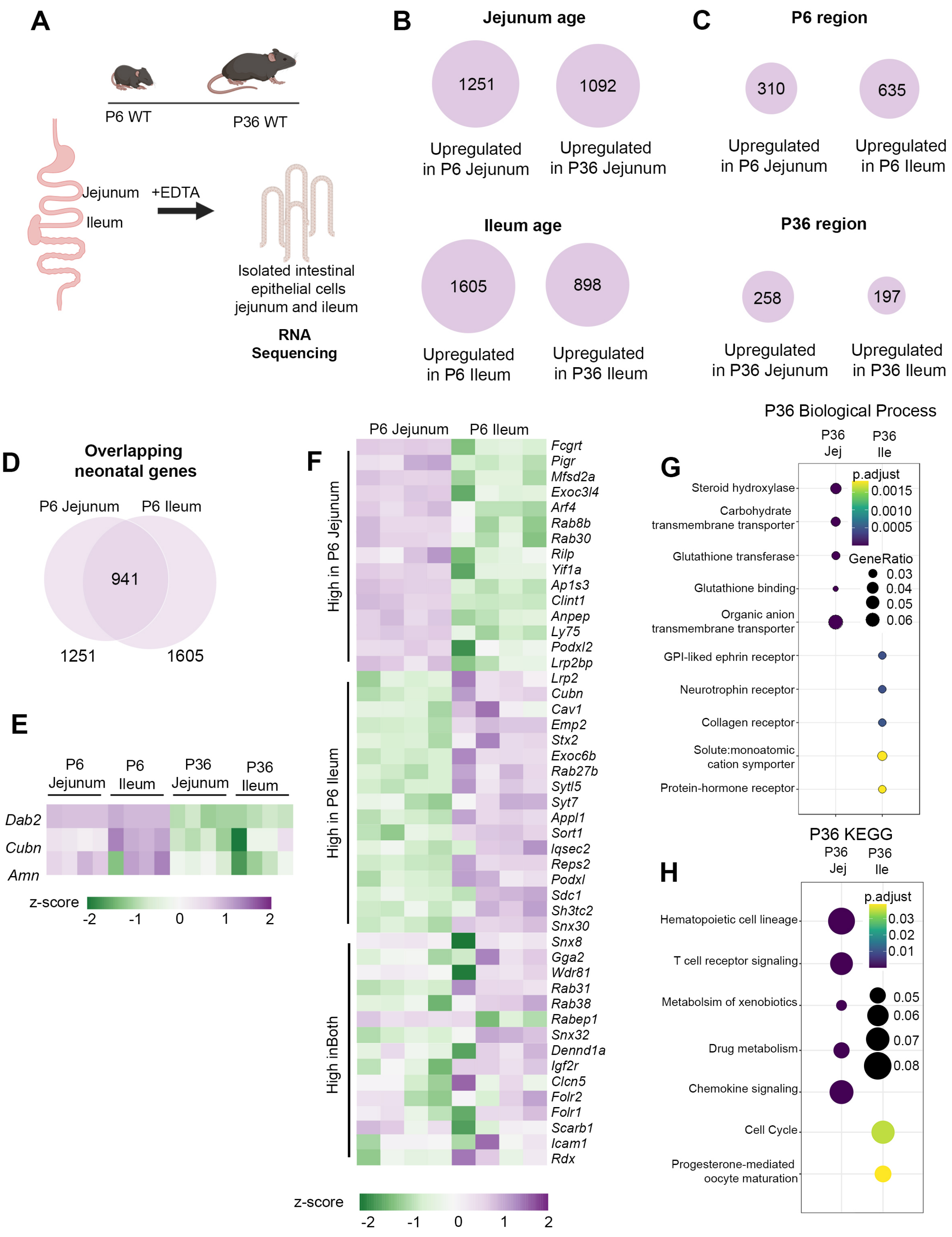

Development • Supplementary information

Fig. S4. Regional differences in gene expression are most robust in neonates

(A) Intestinal segments were incubated with EDTA to release epithelial cells from the intestinal sheet. Created in BioRender by Eroglu, C., 2025. https://BioRender.com/10z33ue. This figure was sublicensed under CC BY 4.0 terms.

(B) Number of differentially expressed genes by age in each region ($Log_2FC > 2$ and p-adjusted <0.05).

(C) Number of differentially expressed genes by region in each age ($Log_2FC > 2$ and p-adjusted <0.05). There are more differentially expressed genes by region at P6.

(D) Genes upregulated by age in both jejunum and ileum, 941 of these genes were upregulated in both regions, displayed by the overlap.

(E) Expression of *Dab2, Cubn* and *Amn* was higher in both the P6 jejunum and ileum compared to P36.

(F) Heat map of endocytic genes in P6 jejunum versus P6 ileum.

(G) Dot plot showing gene ontology for biological process for genes upregulated by region at P36.

(H) Dot plot showing KEGG pathway analysis for genes upregulated by region at P36.

Development • Supplementary information

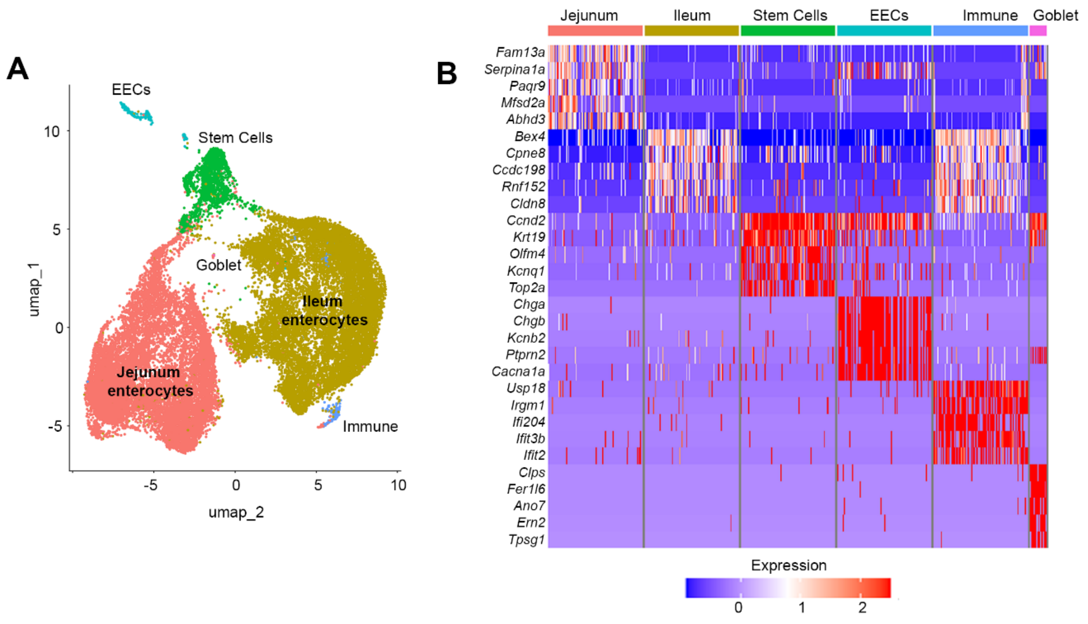

Fig. S5. Cluster Markers

(A) UMAP with all jejunum enterocytes and ileum enterocytes condensed into regional groups

(B) Heat map of scaled data with the top 5 genes enriched in each cluster

Development • Supplementary information

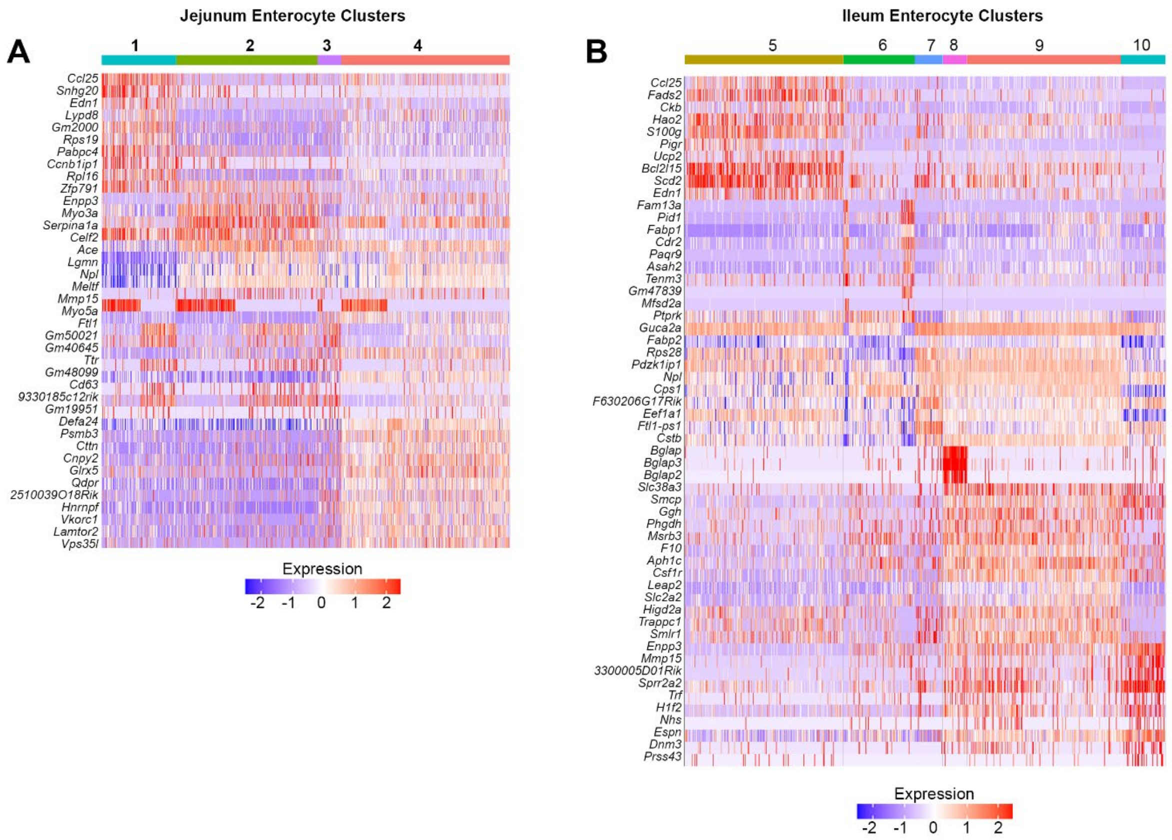

Fig. S6. Enterocyte cluster gene signatures

(A) Heat map of the top genes in the jejunum clusters

(B) Heat map of top genes in the ileum clusters

Development • Supplementary information

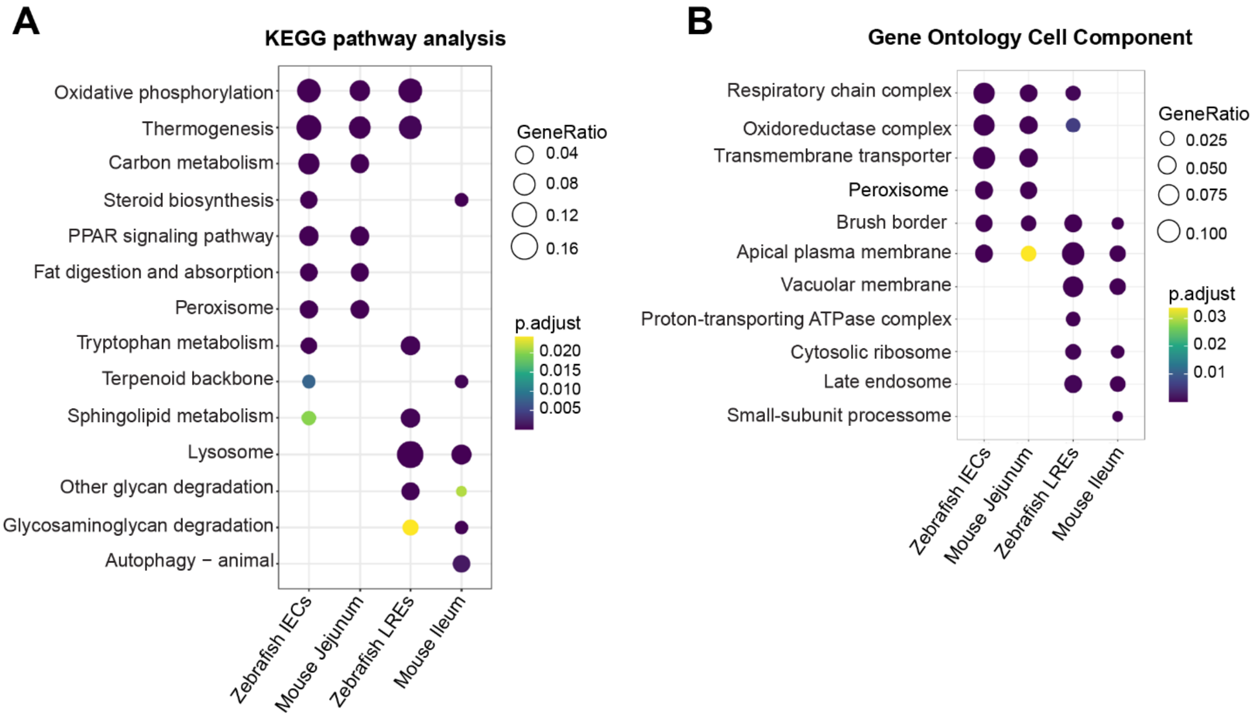

Fig. S7. Cross-species pathway analysis

(A) Dot plot of KEGG pathways significantly enriched in zebrafish IECs and LREs and mouse jejunum and ileum enterocytes.

(B) Dot plot of gene ontology terms for cellular components significantly enriched in zebrafish IECs and LREs and mouse jejunum and ileum enterocytes.

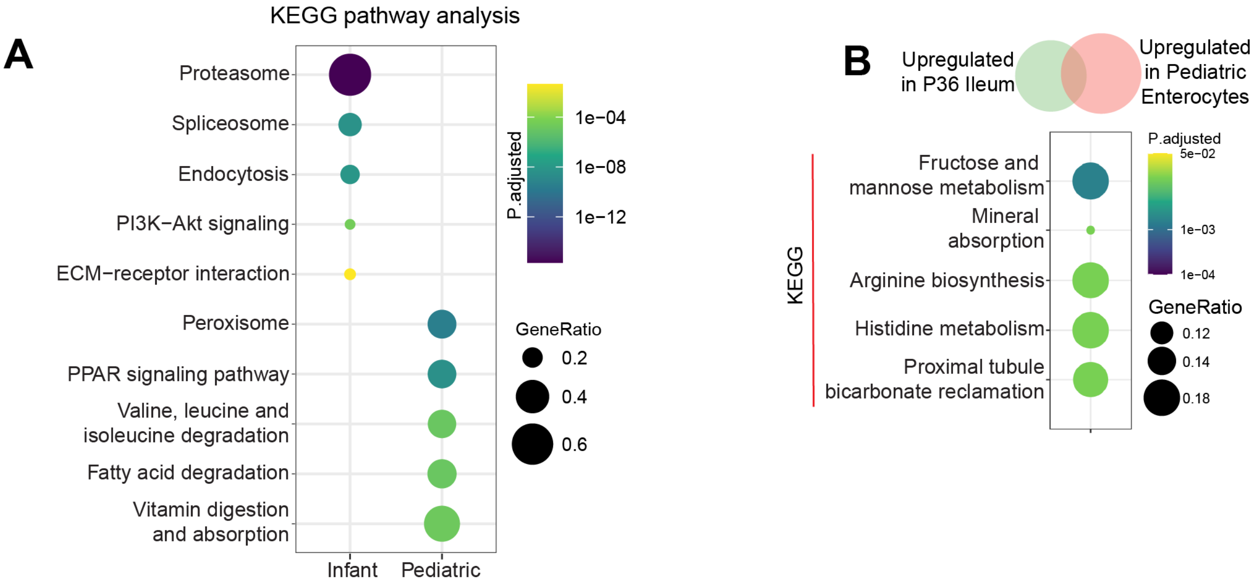

Fig. S8. Pathway analysis in infant and pediatric enterocytes

A) KEGG pathway analysis of upregulated genes in infant and pediatric enterocytes.

B) KEGG pathway analysis was performed on genes overlapping between the P36 mouse ileum and genes upregulated in pediatric enterocytes.

Development • Supplementary information

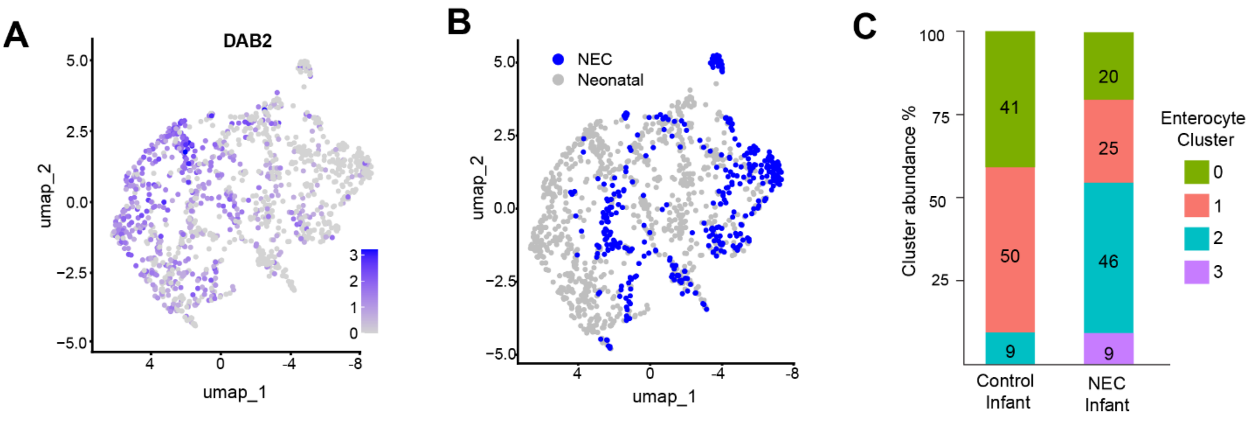

Fig. S9. NEC depletes LRE signature cluster

A) Feature plot of DAB2 expression in infant enterocytes.

B) UMAP of infant enterocytes, highlighting the distribution of NEC infant enterocytes (blue).

C) Cluster abundance was calculated as the percentage of cells in each cluster over the total number of cells per condition.

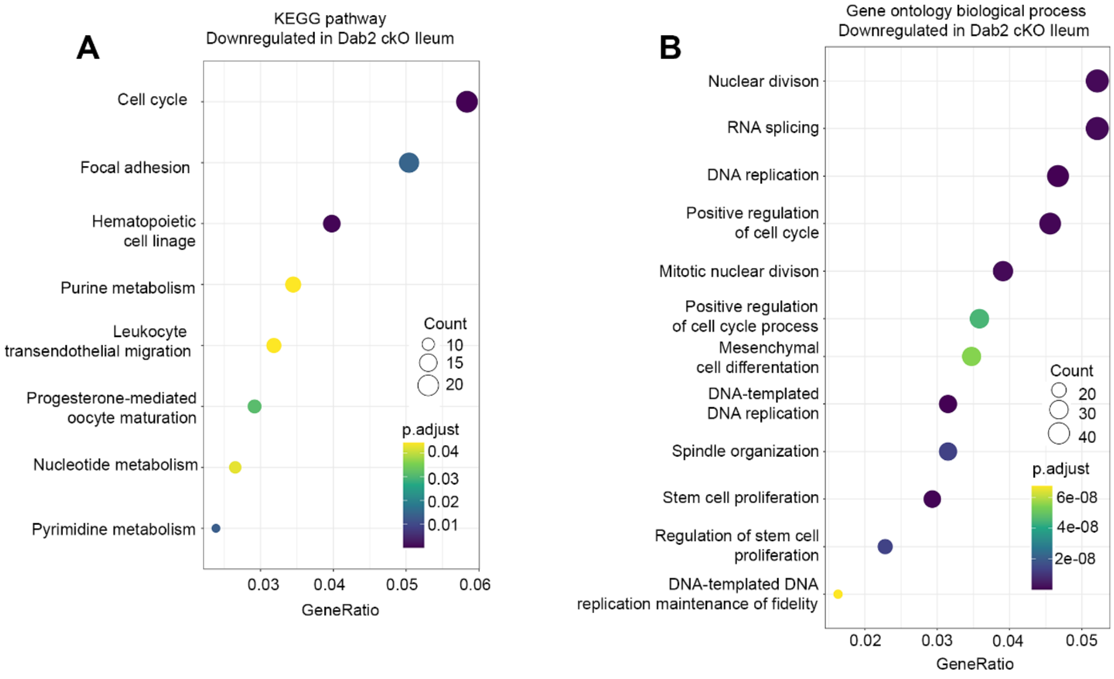

Fig. S10. Downregulation of cell cycle and replication in Dab2 cKO ileum

A) KEGG pathway analysis of downregulated genes in the Dab2 cKO ileum

B) Gene ontology for biological process of downregulated genes in Dab2 cKO ileum

Development • Supplementary information

Table S1. Differentially expressed genes in P6 and P36 jejunum and ileum, related to Fig. 2.

Available for download at
https://journals.biologists.com/dev/article-lookup/doi/10.1242/dev.205127#supplementary-data

Table S2. Single-cell count data, related to Figure 3

Available for download at
https://journals.biologists.com/dev/article-lookup/doi/10.1242/dev.205127#supplementary-data

Table S3. Cluster markers in P6 jejunal enterocytes, related to Fig. 3

Available for download at
https://journals.biologists.com/dev/article-lookup/doi/10.1242/dev.205127#supplementary-data

Table S4. Cluster markers in P6 ileal enterocytes, related to Fig. 3

Available for download at
https://journals.biologists.com/dev/article-lookup/doi/10.1242/dev.205127#supplementary-data

Table S5. Cluster markers in human infant enterocytes, related to Fig. 4

Available for download at
https://journals.biologists.com/dev/article-lookup/doi/10.1242/dev.205127#supplementary-data

Table S6. Differentially expressed genes in P6 Dab2 cWT and cKO jejunum and ileum, related to Fig. 5

Available for download at
https://journals.biologists.com/dev/article-lookup/doi/10.1242/dev.205127#supplementary-data
